# Supplementary material for: Causal Relationship between Aging and Anorexia Nervosa: A White-Matter-Microstructure-Mediated Mendelian Randomization Analysis
Source: Biomedicines. 2024 Aug 16;12(8):1874. doi: 10.3390/biomedicines12081874 (PMC11351342; doi:10.3390/biomedicines12081874)
Supplement: Supplementary file 1 [file biomedicines-12-01874-s001.zip › Supplementary Figure/Figure S8/Leaveoneout plot of MR analysis for TL to ubm-b-1499.pdf]

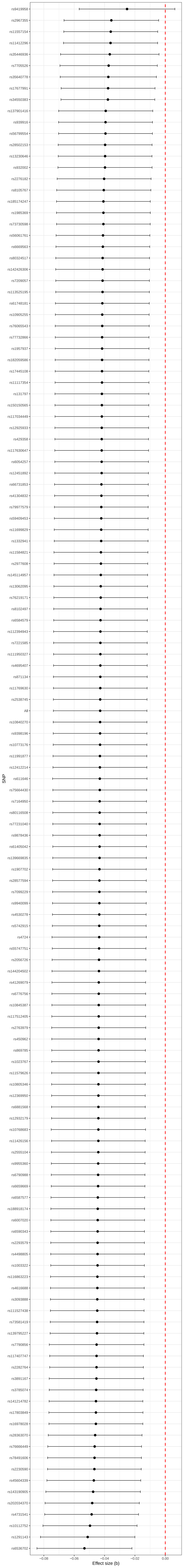
rs9419958
rs2967355
rs11557154
rs11412296
rs35446936
rs7705526
rs35640778
rs17677991
rs34550383
rs137901416
rs939916
rs56799554
rs28502153
rs13230646
rs932002
rs2276182
rs8105767
rs185174247
rs1985369
rs73730598
rs56061761
rs6669563
rs80324517
rs142426306
rs7209057
rs113525195
rs61748181
rs10905255
rs76065543
rs77732866
rs1957937
rs182059586
rs17445108
rs11117354
rs131797
rs150150565
rs117034449
rs12925933
rs429358
rs117630647
rs6054257
rs12451892
rs66731853
rs41304832
rs79977579
rs59409453
rs11699829
rs1332941
rs11584821
rs2977608
rs145114957
rs13062095
rs76219171
rs8102497
rs6584579
rs112394943
rs7221585
rs111950327
rs4695407
rs871134
rs11769630
rs2538745
All
rs10840270
rs9398196
rs10773176
rs11991877
rs12412214
rs611646
rs75664430
rs7164950
rs80116508
rs77231040
rs9878436
rs61405042
rs139669835
rs1907702
rs28577594
rs7099229
rs9940099
rs4530278
rs5742915
rs4724
rs55747751
rs2056726
rs144204502
rs41269079
rs6776756
rs10845387
rs117512405
rs2763979
rs450962
rs869785
rs1023767
rs11579626
rs10805346
rs12369950
rs6881568
rs12932179
rs10768683
rs11426156
rs2555104
rs9955360
rs6790988
rs6659669
rs6587577
rs188918174
rs6007020
rs6590343
rs2293579
rs4498805
rs1003322
rs116863223
rs4616688
rs3093888
rs111527438
rs73581419
rs139795227
rs7790856
rs117407747
rs2282764
rs3891167
rs3785074
rs141214782
rs17803849
rs16978028
rs28363070
rs76666449
rs78491606
rs2230590
rs45604339
rs143190905
rs202034370
rs4731541
rs10112752
rs1291143
rs6536702
SNP
-0.08
-0.06
-0.04
-0.02
0.00
Effect size (b)
